# Effects of coffee pericarp and litter mulsching on soil microbiomes diversity and functions in a tropical coffee plantation, South China

**DOI:** 10.3389/fmicb.2023.1323902

**Published:** 2024-01-08

**Authors:** Shaoguan Zhao, Ang Zhang, Qingyun Zhao, Yaoyu Zhang, Dong Wang, Lanxi Su, Xingjun Lin, Yan Sun, Lin Yan, Xianwen Wang, Na An, Yunping Dong, Jun Tan, Yuzhou Long, Zhiqing Lu, Lihua Li

**Affiliations:** ^1^Spice and Beverage Research Institute of Chinese Academy of Tropical Agricultural Science, Wanning, Hainan, China; ^2^College of Tropical Crop Science, Yunnan Agricultural University, Kunming, Yunnan, China; ^3^School of Life Science, Henan University, Kaifeng, Henan, China; ^4^Yan Lin Expert Workstation of Yunnan Province, Baoshan, Yunnan, China; ^5^Baoshan Comprehensive Inspection Center For Quality Technology Supervision, Baoshan, China; ^6^Dong Yunping Expert Workstation of Yunnan Province, Puer, Yunnan, China

**Keywords:** coffee pericarp, coffee litter, mulch cultivation, soil microbiome, soil health

## Abstract

In recent decades, ecological cyclic cultivation models have attracted increasing attention, primarily because the decomposition of crop residues and litter enhances soil organic matter content, thereby altering the soil microenvironment and regulating the diversity and functions of soil microbial communities. However, the effects of different coffee waste mulching on the diversity of soil microbial communities and their functions are still unclear. Therefore, this study set up four kinds of covering treatments: uncovered coffee waste (C), covered coffee litter (L), covered coffee pericarp (P), and both covered coffee litter and pericarp (PL). The results showed that compared to the control, coffee pericarp mulching significantly increased the soil available potassium (SAK) content by 18.45% and alkali hydrolyzed N (SAN) content by 17.29%. Furthermore, coffee pericarp mulching significantly increased bacterial richness and diversity by 7.75 and 2.79%, respectively, while litter mulching had little effect on bacterial abundance and diversity was smaller. The pericarp mulching significantly increased the abundance of *Proteus* by 22.35% and the abundance of *Chlamydomonas* by 80.04%, but significantly decreased the abundance of *Cyanobacteria* by 68.38%, while the coffee litter mulching significantly increased the abundance of *Chlamydomonas* by 48.28%, but significantly decreased the abundance of *Cyanobacteria* by 73.98%. The increase in soil SAK promoted bacterial Anoxygenic_photoautotrophy, Nitrogen_respiration, Nitrate_respiration, Nitrite_respiration, and Denitrification functions. The above results indicate that the increase in available soil potassium and alkali hydrolyzed N content under coffee pericarp cover is the main reason for promoting the diversity and richness of bacterial community and promoting the changes in bacterial community structure and function. The use of coffee pericarps in coffee plantations for ecological recycling helps to improve the diversity of the soil microbial community and maintain the relative stability of the microbial community structure and function, promoting soil health conservation and the sustainable development of related industries.

## 1 Introduction

Coffee is one of the main trade commodities in the world, with an annual consumption of approximately 10 million tons (Chain-Guadarrama et al., 2019). It plays a crucial role in promoting the development of the global economy (Duran-Aranguren et al., 2021). Coffee production is mainly concentrated in developing countries located in tropical areas. The cost savings and increased efficiency in coffee production contribute to agricultural development and poverty alleviation among farmers in these countries (Silva et al., 2020). Traditionally, coffee is cultivated using a mulching process, which enhances soil fertility, increases organic matter content, and creates a favorable microenvironment for root development (Jian et al., 2020; Rosado et al., 2021), This cultivation method also helps inhibit weed competition and promotes nutrient absorption, ultimately leading to higher coffee yields (Stewart et al., 2018; Zhang et al., 2022). However, the accumulation of by-products, such as coffee pericarp from primary processing, poses environmental pollution challenges, hindering the sustainable development of the coffee industry (Dos Santos et al., 2021). To address this issue, an environmentally friendly cultivation approach that involves recycling coffee litter and pericarps instead of traditional mulch has been proposed (Duran-Aranguren et al., 2021). This approach not only reduces the production cost of coffee cultivation but also promotes the recycling of coffee waste resources, improving overall coffee utilization. As a result, it has the potential for large-scale implementation.

Soil health is the basis for promoting efficiency in coffee production, but the impact of coffee ecological recycling cultivation systems on soil health is not clear at present. As an important component of soil ecosystems, soil microbiome diversity is one of the key factors driving biogeochemical cycles of carbon, nitrogen, sulfur, and phosphorus (Rahman et al., 2021), which has become an important indicator of soil health in agroecosystems (Sapkota et al., 2021). Previous studies have shown that the physicochemical and biological properties of soil ecosystems are the main factors influencing the diversity of microorganisms (Wang et al., 2020). Coffee ecological recycling cultivation systems can increase the diversity and richness of the soil microbial community by enhancing soil moisture content, enhancing soil biological activity, and promoting soil mineralization and accumulation of carbon, nitrogen, and phosphorus (Muhammad et al., 2021; Liu et al., 2023). For example, straw mulching increases the diversity and richness of the rhizosphere bacterial community by increasing the soil organic carbon (SOC) content and providing an effective carbon source for bacteria (Chen et al., 2022). The mixed mulch of plastic film and straw has either a positive or negative effect on the diversity of bacteria, actinobacteria, and fungi by changing the soil temperature (Li et al., 2016). The increase in soil moisture in the mulching mode usually contributes to improving microbial activity, which has a positive feedback effect on microbial diversity by stimulating soil biological activity (Gao et al., 2022; Li et al., 2022). Furthermore, the decomposition of plant-derived mulch is closely related to soil microbiome assembly (Jose Fernandez-Alonso et al., 2022). The continuous supply of decomposing substrate not only improves the ecological niche of the soil system and contributes to an increase in microbial diversity, but also promotes the growth of beneficial rhizosphere bacteria *Bacillus* and *Pseudomonas* by affecting the metabolic activities of different microbial taxa (Wang L. et al., 2022), and alleviating the intensity of interspecific competition, and then improves the soil microbial diversity (Gao et al., 2022).

There is a significant coupling relationship between soil microbiome structure assembly and diversity (Zosso et al., 2021). The change in cultivation patterns can significantly change the structure of the soil microbiome through four regulatory pathways in the ecological recycling cultivation system (Gao et al., 2022; Wang Y. et al., 2022; Zhang M. et al., 2023). First, the mulching of exogenous agricultural waste promotes the recruitment of more strains of colonized soil *in situ*, which is advantageous to accelerate microbial community succession and thus change the microbial community structure (Sun et al., 2022). Second, mulch directly regulates the structure of the soil microbiome mainly by changing the microenvironment of the farmland (Muhammad et al., 2021) and the soil nutrient content (Zheng et al., 2022). For example, mulch reshapes soil microbiome structures and networks by altering soil pH, temperature, and soil water content, and provides additional organic substrate by affecting plant residue accumulation and rhizodeposition (Castellano-Hinojosa and Strauss, 2020), regulating bacterial abundance related to soil quality and health (Ferreira Leite et al., 2021). Third, the input of exogenous organic matter in the ecological recycling cultivation system can significantly enhance the cascade effect of the food web (Su et al., 2021), thus promoting the network structure of the soil microbiome and metabolic pathways related to soil nutrient cycling, and forming in a more stable exobiotic community structure (Castellano-Hinojosa and Strauss, 2020). Fourth, soil microbial structure is closely related to soil enzyme activity. Mulch significantly affects soil microbial community structure by changing the interaction between soil microecological environment-nutrient availability-soil microbial community structure (Muhammad et al., 2021; Gao et al., 2022). Moreover, exogenous mulch addition may cause significant separation of soil bacterial and fungal composition (Zhang S. et al., 2023), due to the different physiological functions of bacteria and fungi in soil and their response to mulch (Drost et al., 2020).

Previous studies have suggested that agricultural mulch diversity and properties are the important reasons for the differences in the soil microbial community structure (Jabiol et al., 2019). For example, compared to the leaf litter, coffee pericarp contains a higher ratio of carbon and nitrogen (Shemekite et al., 2014; Du et al., 2021), and leaf litter with higher nitrogen content has faster decomposition rates in the early stages of coffee mulch decomposition (Wang et al., 2021). The differences in decomposition products affect the soil microbiome by significantly changing soil physicochemical properties (Yan et al., 2018), and the changes in the soil microbiome in turn have a feedback effect on the decomposition mechanisms of litter and pericarp (Dong et al., 2021). The previous study concluded that there is an obvious matching relationship between soil microbial functions and their community structure (Waring et al., 2022). For example, a long-term ditch-buried straw return increased the soil fungal evenness and diversity by improving soil microbiome functions such as the secretion of enzymes involved in regulating soil carbon, nitrogen, sulfur, and phosphorus cycling (Yang et al., 2021). Furthermore, soil microenvironments such as soil temperature, humidity, substrate availability, and nutrient limitation can also affect soil carbon metabolism, including functions such as microbial growth, carbon use efficiency, respiration, and microbial biomass turnover (Zheng et al., 2019). Therefore, the effects of different coffee mulches on the structure and function of soil microbiome need to be further investigated. This study proposes to solve the following scientific questions through controlled field experiments: (1) Clarify the diversity and functional differences of soil microbiome and its key driving factors under coffee litter and pericarp mulching; (2) Explore the dynamic balance mechanism of soil microbiome structure and function regulated by coffee litter and pericarp decomposition. The results of the study could provide a theoretical basis for optimizing coffee ecological recycling cultivation technology and maintaining the sustainable development of ecosystem service functions in coffee plantations.

## 2 Materials and methods

### 2.1 Experimental site

The experiment was located in the coffee germplasm resource nursery of Xinglong Tropical Botanical Garden, Hainan Province, China (110°11′ E, 18°44′ N, 36 m a. s. l.). The experimental site is located near the eastern coast of Hainan Island, which belongs to the tropical monsoon climate. The annual precipitation was 2,100 mm and the annual sunshine duration exceeds 1,750 h. The soil was mainly classified as tidal sand-mud (US Soil Taxonomy classification). Soil pH was 6.15, soil organic matter content (SOM) was 22.06 g kg^−1^, soil total nitrogen content (STN) was 1.49 g kg^−1^, soil total phosphorus content (STP) was 1.28 g kg^−1^, and soil total potassium content (STK) was 6.29 g kg^−1^. The coffee variety used in this experiment is Reyan No.5 medium-grain coffee.

### 2.2 Experimental design

The experiment was set up in September 2020. A randomized block design was conducted with four mulching treatments: uncovered coffee waste (C), covered coffee litter (L), covered coffee pericarp (P), and covered coffee litter and pericarp (PL) simultaneously. Each block was repeated four times. A random survey of the cumulative amount of existing coffee litter and pericarp in coffee plantations was 301.39 g m^−2^ and 239.05 g m^−2^, respectively. The litter and pericarp were added equivalently to the average cumulative amount. To maintain optimal soil moisture levels, the coffee plants were irrigated once every week with precise amounts of water during each irrigation session, ensuring that the soil moisture reached a specific level (e.g., 50% water capacity). The conventional fertilization method was implemented for both the experimental and control groups, guaranteeing that all coffee plants received the necessary nutrients in line with their specific requirements. It is worth noting that the same coffee variety and planting environment were utilized across all the experimental and control groups.

### 2.3 Sampling method

The soil samples were collected with a soil auger in September 2021 and all sampling points were randomly selected. Three original soil samples at 0-20 cm were mixed as one soil sample. A part of the mixed soil sample was dried in the shade and then screened through a 1 mm sieve for the determination of soil physicochemical properties. Another part of the mixed soil samples was immediately stored in the −80°C refrigerator for the determination of soil microbiomes.

### 2.4 Samples analysis

Soil moisture (SM) was assessed by oven drying to a constant mass at 75°C for 48 h. Soil temperature (ST) was measured by the thermocouple probe, which connected to the portable soil carbon dioxide flux measurement system (Li-8100, Li-Cor, Inc., Lincoln, NE, USA). Soil bulk density (SBD) was calculated by the gravimetric weight with a ring knife. Soil pH was measured by an FE28 pH meter in a 1:2.5 soil/water suspension solution. Soil organic matter (SOM) was measured by a total organic carbon analyzer (Multi N/C 3100, Jena, Germany). Soil alkali hydrolyzed Nitrogen (SAN) was determined by the alkaline digestion and diffusion method. Soil available phosphorus (SAP) was determined by the Bray method (UV2310 II, Shanghai, China). Soil available potassium (SAK) was determined by the flame photometer method (6400A, Changsha, China) (Zhong et al., 2022).

### 2.5 Extraction and sequencing of soil total DNA

Soil microbial DNA was extracted three times from 0.5 g fresh soil using the EZNA® Soil DNA Extraction Kit (Omega, USA). A 0.8% agarose gel was used to check the purity and quality of genomic DNA. Barcode-labeled primer sequences for bacteria, 338F (5′-ACTCCTACGGGAGGCAGCAG-3′) and 806R (5′-GGACTACHVGGGTWTCTAAT-3′), and fungi, ITS1F (5′-CTTGGTCATTTAGAGGAAGTAA-3′) and ITS2R (5′-GCTGCGTTCTTCATCGATGC-3′), were used to amplify the corresponding soil bacterial 16S rRNA V3-4 region fragment and fungal ITS-1 sequence fragment. The length of the amplified product fragments was detected on 2% agarose gel electrophoresis. The amplified products were mixed into one sample, and then a clone library was constructed, according to the quantitative detection results. The loading amount for each library was calculated based on the library search results, and the paired-end sequencing method was used on the Illumina MiSeq high-throughput platform for sequencing. The loading amount of each library was calculated, and the paired-end sequencing method was used to utilize Illumina MiSeq, according to the library inspection results.

### 2.6 Bioinformatics analysis

FLASH 1.2.11 software and quality filtering using QIIME 1.9.1 software were used to obtain valid sequences by merging paired-end reads of raw DNA fragments. Unique sequences with 97% or greater similarity were clustered into operational taxonomic units (OTUs) using UPARSE 7.0.1090 software. Each OTU using the small subunit rRNA SILVA database was annotated by MOTHUR 1.30.2, and the sample with the least data was used as the standard for normalization. Soil microbial community richness and diversity were calculated using QIIME 1.9.1 software.

### 2.7 Data analysis

Two-way analysis of variance (Two-way ANOVA) was used to determine the difference between the effect of coffee pericarp and litter mulch patterns on soil properties and microbial characteristics (i.e., ST, SM, SBD, pH, SOM, SAK, SAP, SAN, bacterial richness and diversity, fungal richness and diversity, and functions profiles of soil bacteria and fungi). Before the data analysis, all the data were in accord with the normality test. Data analysis was performed using the Duncan test, and the difference between mean values was determined using the least significant difference (LSD).

The Shannon index was calculated as the estimated taxonomic alpha diversity of bacteria and fungi community using the MOTHER v.1.33.3 software, respectively. Nonmetric multidimensional scaling analysis (NMDS) was executed to illustrate the clustering of different samples and further reflect the bacteria and fungi community structure, while the changes in microbial structure under several mulch patterns were referred to as the beta diversity of bacteria and fungi, respectively. Redundancy analysis (RDA) was performed to analyze the relation between microbial (i.e., bacteria and fungi) characteristics (i.e., richness and diversity) and soil properties using the CANOCO 4.5 software package. The model was assessed for 999 iterations based on Monte Carlo permutations. The graphs were plotted using Origin 2021b and R.4.0.5. The correlations between the microbial (i.e., bacteria and fungi) composition and the soil properties were determined by Spearman’s correlation analysis (SPSS 23.0, SPSS Inc., Chicago, USA). Network interaction analysis between soil properties and microbial (i.e., bacteria and fungi) composition was analyzed and painted by Cytoscape V3.8.2. FAPROTAX (Liang et al., 2020) and FUNGuild (Jiang et al., 2021) are commonly used to predict the functions of bacterial and fungal communities.

## 3 Results

### 3.1 Effects of coffee pericarp and litter mulching on soil physicochemical properties and micro-environment

**Figure 1:**
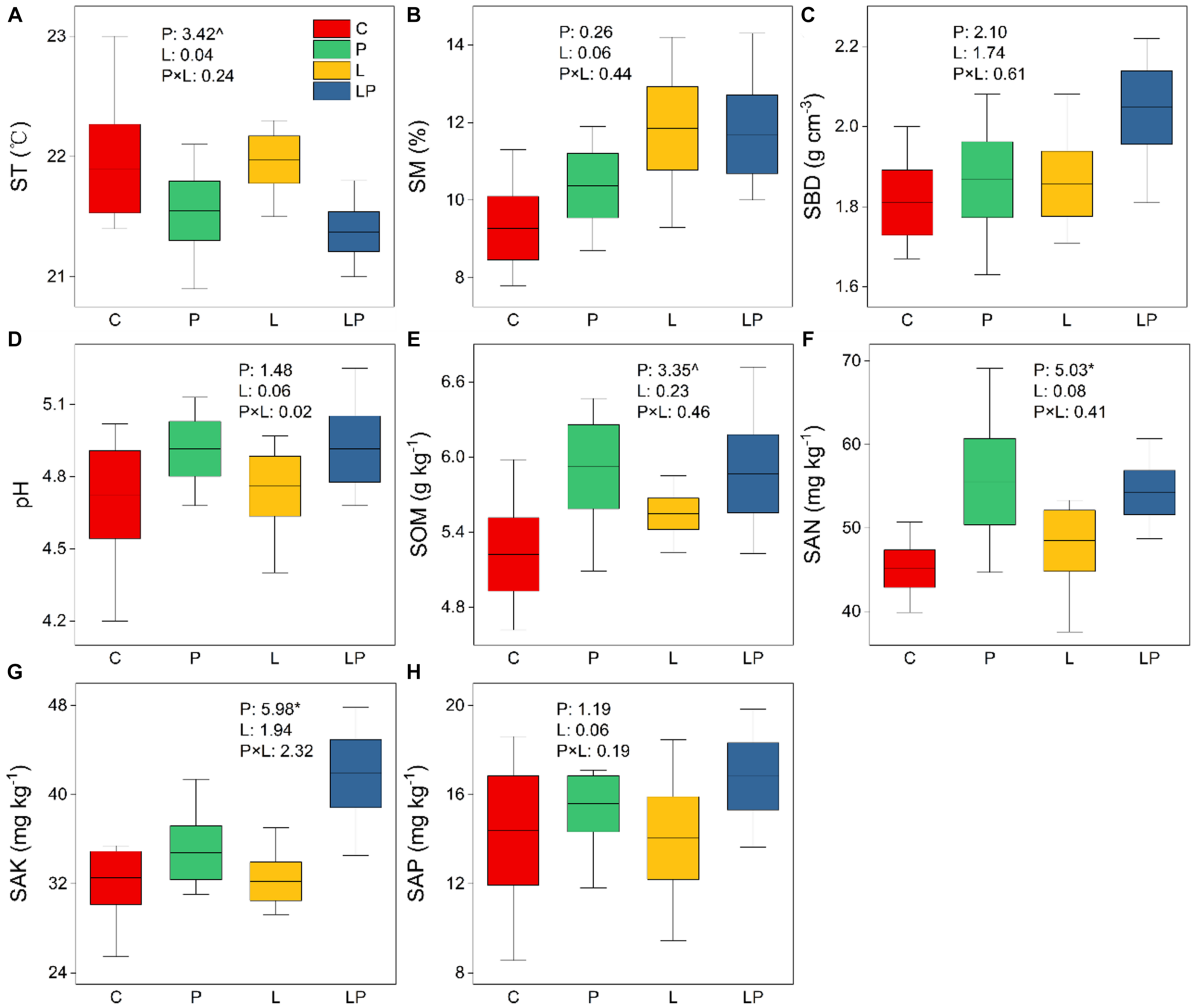
Physicochemical properties of the soil under coffee pericarp and litter mulch patterns. C represent the control treatments, P represent coffee pericarp mulch, L represent coffee litter mulch, PL represent coffee pericarp and litter mulch; **(A)** Soil temperature, **(B)** Soil moisture, **(C)** Soil bulk density, **(D)** pH value, **(E)** Soil organic matter, **(F)** Soil alkali hydrolyzed Nitrogen, **(G)** Soil available phosphorus, **(H)** Soil available potassium; Significant level: “^” indicate *p* < 0.1; “*” indicate *p* < 0.05; “**” indicate *p* < 0.01; “***” indicate *p* < 0.001.

The results of two-way ANOVA showed that coffee pericarp mulching significantly increased the content of SAN and SAK by 17.29 and 18.45% (both *p* < 0.05), respectively, and it had a tendency to inhibit and stimulate the content of ST and SOM (*p* < 0.1), respectively. However, coffee litter mulching had no effect on SAN, SAK, ST, or SOM, and there was no interaction effect between coffee pericarp and litter mulching on these indicators. Coffee pericarp and litter mulching had no effects on SM, SBD, pH, and SAP, and there was also no interaction effect between coffee pericarp and litter mulching on these indicators (Figure 1).

### 3.2 Effects of coffee pericarp and litter mulching on soil microbial richness and diversity

**Figure 2:**
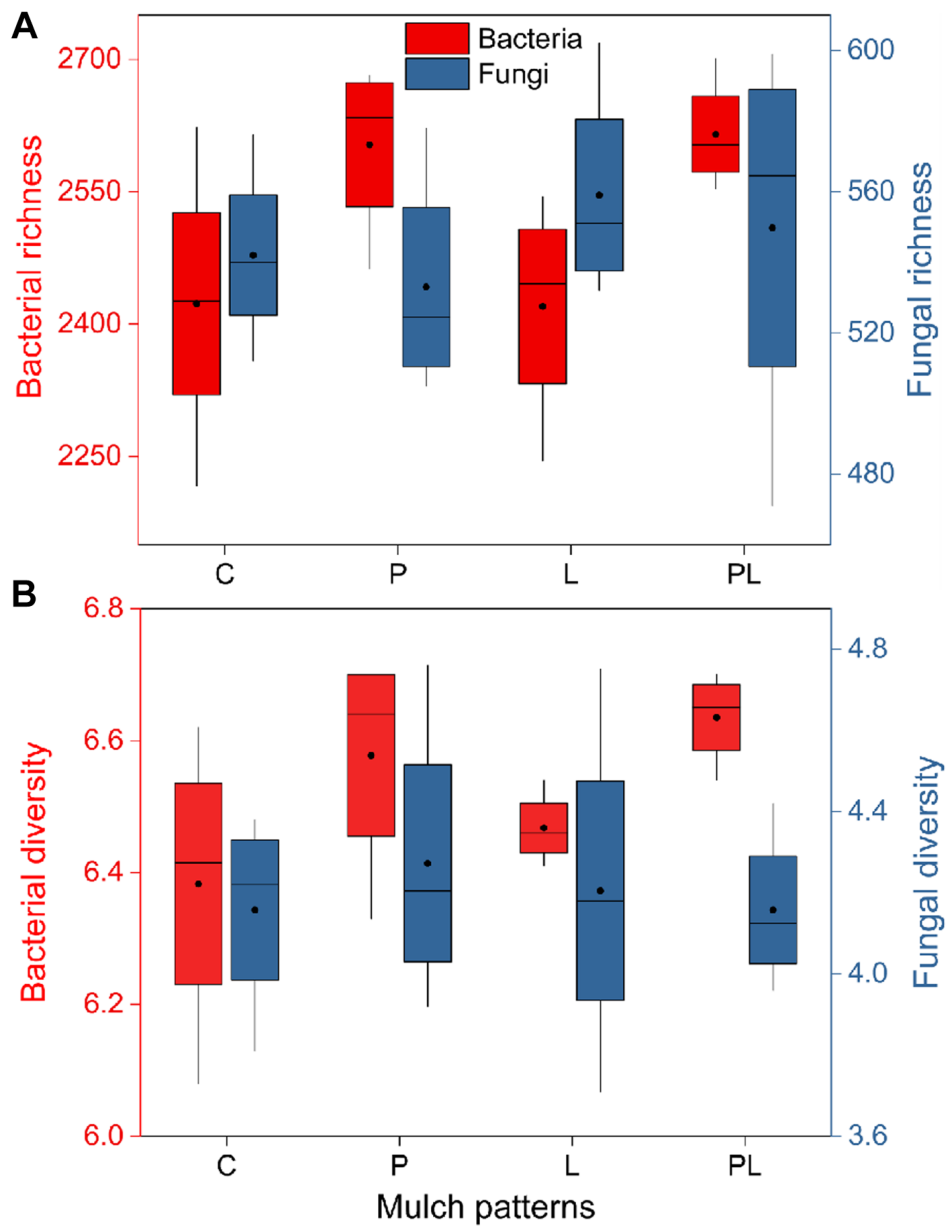
Effects of coffee pericarp and litter mulch patterns on soil microbial (bacterial and fungal) richness **(A)** and alpha diversity [Shannon index, **(B)**]. See Figure 1 for treatment abbreviations.

**Figure 3:**
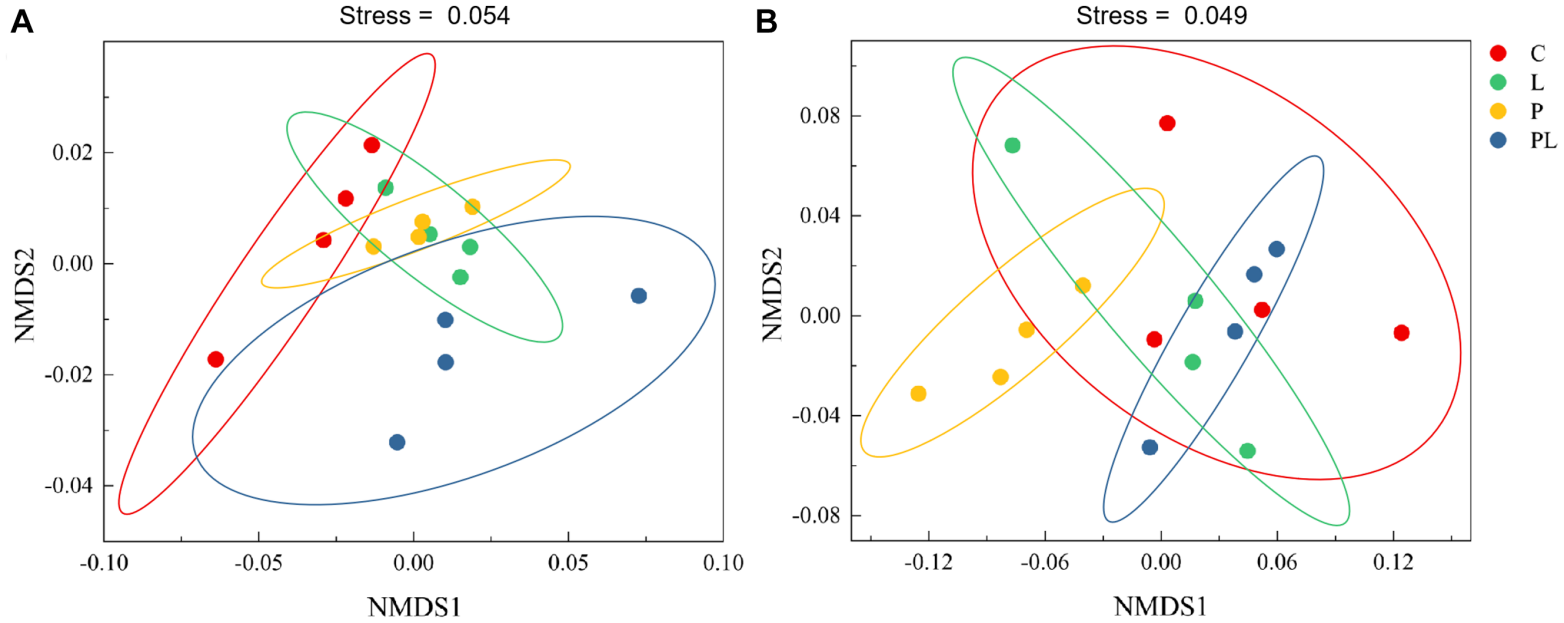
Effects of coffee pericarp and litter mulch patterns on soil bacterial **(A)** and fungal **(B)** beta diversity (NMDS) across the experimental period. See Figure 1 for treatment abbreviations.

The results of a two-way ANOVA showed that coffee pericarp mulching significantly increased the bacterial richness (Richness index) and diversity (Shannon index) by 7.75 and 2.79% (*p* < 0.05), respectively, while litter mulching had little impact on the bacterial richness and diversity. There was no interaction between coffee pericarp and litter mulching on bacterial richness and diversity (Table 1; Figure 2A). The fungal richness and diversity did not respond to coffee pericarp and litter mulching (Table 1; Figure 2B). Non-metric multidimensional scaling (NMDS) analysis was used to calculate the β diversity of bacterial and fungal communities, respectively, under coffee pericarp and litter mulching. The stress values of bacteria and fungi in the NMDS analysis were 0.054 and 0.049, respectively, indicating that the graph accurately reflected the true distribution of bacterial and fungal communities in the current study (Figure 3). The results showed that compared with the control treatment, the bacterial and fungal β diversity was not affected under the coffee pericarp and litter mulching, despite the β diversity of the bacterial and fungal communities having significant differences between litter mulching and litter and pericarp mulching (Figure 3).

**Table 1:** Results (*F*-values) of repeated measures ANOVAs on the effects of coffee pericarp (P), litter (L) mulch patterns and their potential interactions (P × L) on soil microbial (bacterial and fungal) richness and alpha diversity (Shannon index).

Mulch patterns	Bacterial richness	Bacterial Shannon	Fungi richness	Fungi Shannon
P	9.79^**^	5.9^*^	0.23	0.04
L	0.01	0.91	0.78	0.04
P × L	0.02	0.03	0.01	0.26

Significant level: “^” indicate *p* < 0.1; “*” indicate *p* < 0.05; “**” indicate *p* < 0.01; “***” indicate *p* < 0.001.

### 3.3 Effects of coffee pericarp and litter mulching on soil microbial structure

**Figure 4:**
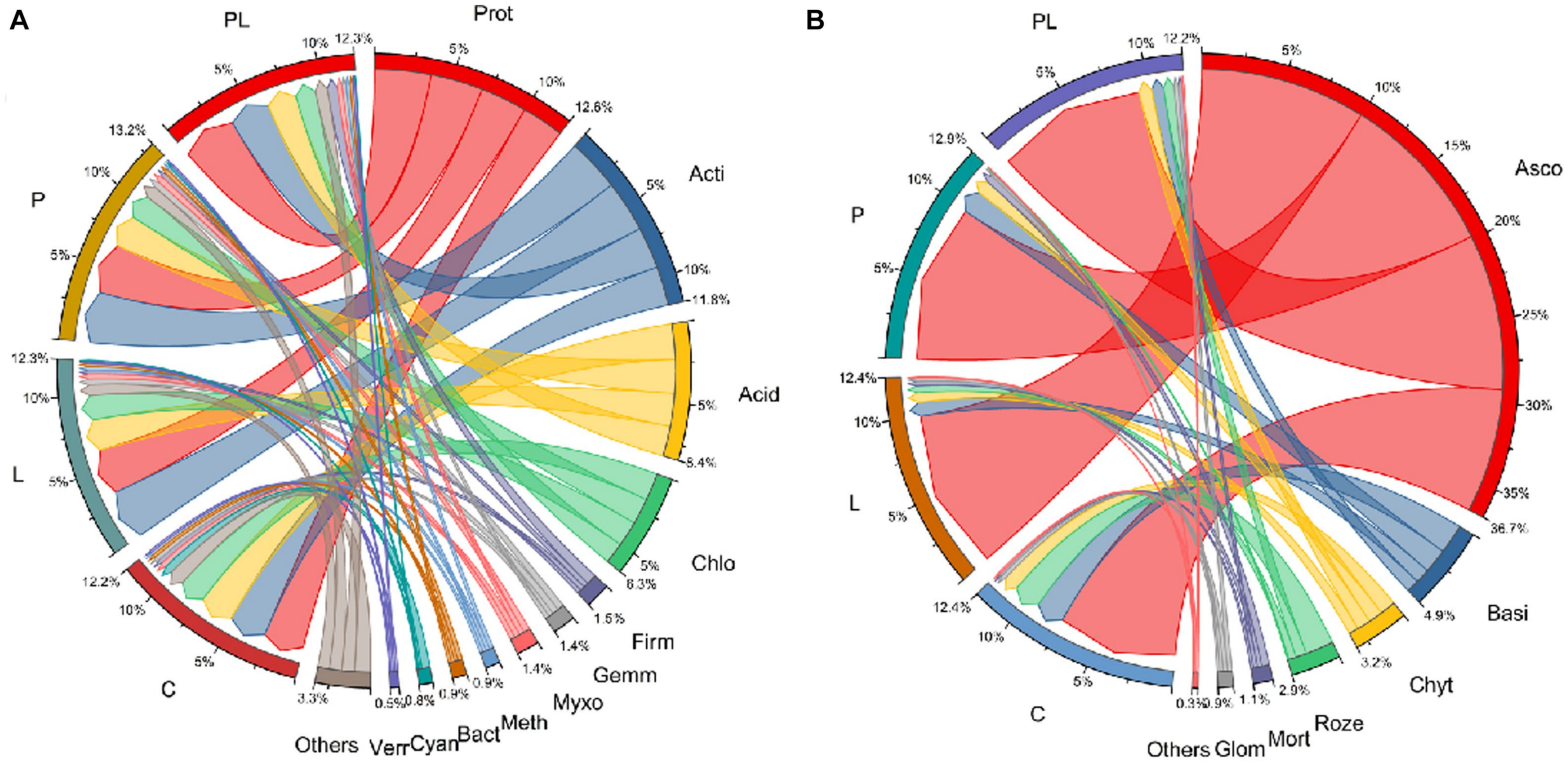
Circular representation of the proportional structure of bacterial **(A)** and fungal **(B)** communities at the phylum level associated with the coffee pericarp and litter mulch patterns. See Figure 1 for treatment abbreviations. Taxa with a proportion lower than 0.1% in all samples are summarized as “Others.” Prot, *Proteobacteria*; Acti, *Actinobacteriota*; Acid, *Acidobacteriota*; Chlo, *Chloroflexi*; Firm, *Firmicutes*; Gemm, *Gemmatimonadota*; Myxo, *Myxococcota*; Myxo, *Methylomirabilota*; Bact, *Bacteroidota*; Cyan, *Cyanobacteria*; Verr, *Verrucomicrobiota*; Asco, *Ascomycota*; Basi, *Basidiomycota*; Chyt, *Chytridiomycota*; Roze, *Rozellomycota*; Mort, *Mortierellomycota*; Glom, *Glomeromycota*.

A microbiome with a relative abundance greater than 1% was defined as the dominant microbes. For bacterial communities, *Proteobacteria*, *Actinobacteriota*, *Acidobacteriota*, *Chloroflexi*, *Firmicutes*, *Gemmatimonadota*, *Myxococcota*, *Methylomirabilota*, *Bacteroidota*, *Cyanobacteria,* and *Verrucomicrobiota* were the dominant bacteria in the bacterial communities in several mulching treatments. Coffee pericarp mulching significantly increased the abundance of *Proteobacteria* and *Firmicutes* by 22.35 and 80.04%, respectively, whereas it significantly decreased the abundance of *Cyanobacteria* by 68.38% (*p* < 0.05, Table 2; Figure 4A). Coffee litter mulching significantly increased the abundance of *Firmicutes* by 48.28% and decreased *Cyanobacteria* by 73.98% (*p* < 0.05). Moreover, coffee pericarp and litter mulching had an interaction effect on *Actinobacteriota*, *Firmicutes*, *Gemmatimonadota*, and *Cyanobacteria* (*p* < 0.05, Table 2; Figure 4A). There was no significant difference in the abundance of *Actinobacteriota*, *Acidobacteriota*, *Gemmatimonadota*, *Myxococcota*, *Methylomirabilota*, *Bacteroidota,* and *Verrucomicrobiota* under several coffee mulch treatments.

**Table 2:** Soil microbial (bacteria and fungi) phylum under coffee pericarp and litter mulch patterns and results (*F*-values) of repeated measures ANOVAs on the effects of coffee pericarp (P), litter (L) mulch patterns and their potential interactions (P × L) on soil microbial (bacterial and fungal) composition.

Functional groups	Microbial phylum	Mulch patterns				*F*-values		
		C	P	L	PL	P	L	P × L
Bacteria	Proteobacteria	6,160 ± 347	7,594 ± 298	6,818 ± 245	8,286 ± 420	7.08^*^	4.46^^^	0.79
	Actinobacteriota	5,713 ± 401	7,749 ± 759	7,517 ± 495	6,067 ± 343	0.01	0.41	6.68^*^
	Acidobacteriota	5,163 ± 538	4,910 ± 354	4,758 ± 722	4,398 ± 1,120	0.67	0.37	0.01
	Chloroflexi	4,022 ± 579	3,766 ± 93	3,693 ± 130	3,056 ± 450	3.52^^^	1.52	0.11
	Firmicutes	689 ± 141	737 ± 61	575 ± 59	1,539 ± 196	9.70^**^	5.91^*^	9.63^**^
	Gemmatimonadota	662 ± 86	975 ± 40	931 ± 60	629 ± 68	0.27	0.04	16.77^**^
	Myxococcota	766 ± 101	912 ± 47	713 ± 35	767 ± 49	0.93	1.17	0.01
	Methylomirabilota	443 ± 70	502 ± 39	550 ± 97	572 ± 78	0.09	2.02	0.01
	Bacteroidota	473 ± 44	637 ± 51	466 ± 22	465 ± 3	2.21	3.00	1.73
	Cyanobacteria	1,221 ± 220	320 ± 37	254 ± 53	147 ± 30	25.54^***^	29.35^***^	15.14^**^
	Verrucomicrobiota	356 ± 42	307 ± 57	255 ± 15	332 ± 115	0.01	0.32	1.06
	Others	2,278 ± 183	1847 ± 134	1,653 ± 34	1849 ± 256	2.11	3.83^^^	7.35^*^
Fungi	Ascomycota	42,049 ± 3,129	55,526 ± 1,250	56,194 ± 2,873	52,782 ± 1,380	6.53^*^	5.27^*^	12.96^**^
	Basidiomycota	10,085 ± 4,039	8,240 ± 1,505	4,964 ± 1,013	4,314 ± 596	3.93^^^	0.03	0.05
	Chytridiomycota	5,351 ± 812	4,765 ± 585	3,391 ± 502	4,597 ± 351	3.48^^^	0.76	2.79
	Rozellomycota	9,580 ± 5,721	484 ± 77	2,474 ± 935	3,612 ± 622	0.89	1.89	7.50^*^
	Mortierellomycota	933 ± 407	2,515 ± 517	1,520 ± 642	1,372 ± 523	1.96	0.19	2.33
	Glomeromycota	1,431 ± 325	850 ± 151	1,147 ± 125	1,537 ± 348	0.19	0.73	3.81^^^
	Other	677 ± 161	136 ± 57	415 ± 127	705 ± 165	0.94	1.49	10.30^**^

C represent the control treatments, P represent coffee pericarp mulch, L represent coffee litter mulch, PL represent coffee pericarp and litter mulch; Significant level: “^” indicate *p* < 0.1; “*” indicate *p* < 0.05; “**” indicate *p* < 0.01; “***” indicate *p* < 0.001.

For fungal communities, *Ascomycota*, *Basidiomycota*, *Chytridiomycota*, *Rozellomycota*, *Mortierellomycota,* and *Glomeromycota* acted as the dominant fungi in this study. Coffee pericarp and litter mulch significantly increased the abundance of *Ascomycota* by 11.68 and 10.25% (*p* < 0.05), respectively. There was an interaction between the effects of coffee pericarp and litter mulch on *Ascomycota* and *Rozellomycota* (*p* < 0.05, Table 2; Figure 4B). There is no difference in the abundance of *Basidiomycota*, *Chytridiomycota*, *Rozellomycota*, *Mortierellomycota,* and *Glomeromycota* under several coffee mulch treatments (Table 2; Figure 4B).

### 3.4 Effects of coffee pericarp and litter mulching on soil microbial group functions

**Figure 5:**
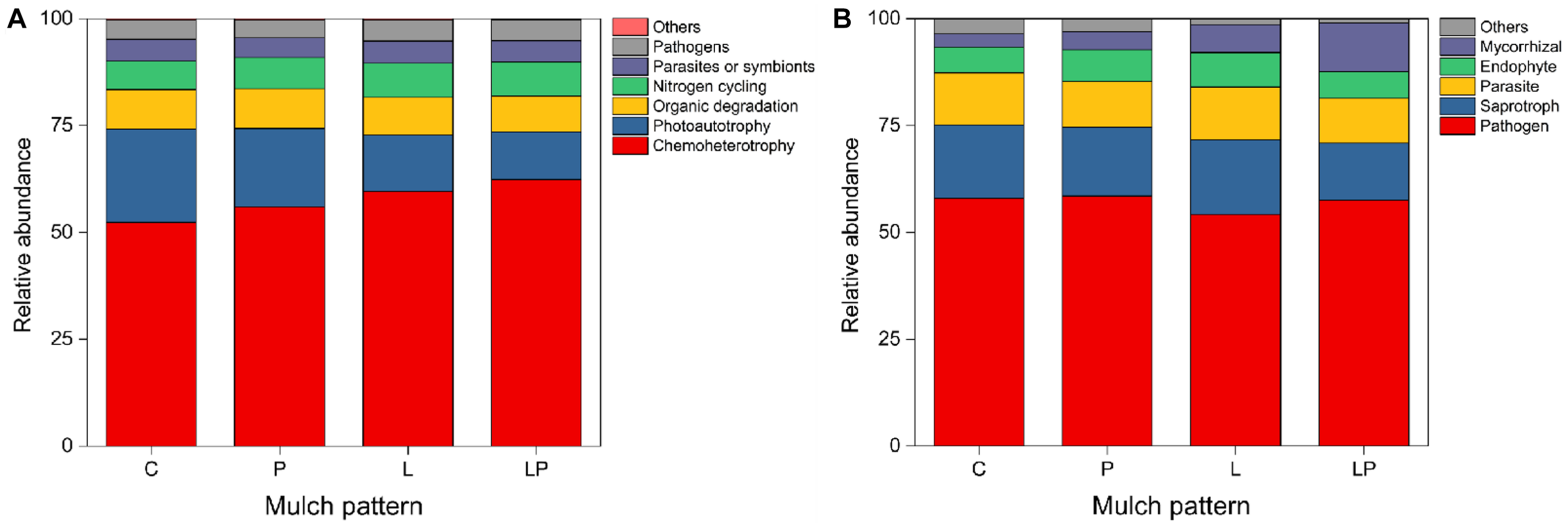
Predicted functional profiles of the soil bacteria **(A)** and fungi **(B)** under coffee pericarp and litter mulch patterns. See Figure 1 for treatment abbreviations.

For the functions of bacteria, coffee litter mulching significantly increased chemotherotrophy and the nitrogen cycle by 11.28 and 4.83%, respectively, but significantly reduced photoautotrophy and organic degradation by 13.33 and 1.95%, respectively (*p* < 0.05, Figure 5A). Similarly, coffee pericarp mulching also significantly increased chemotherotrophy and the nitrogen cycle by 10.05 and 4.69%, respectively, but significantly reduced photoautotrophy by 12.79% in the current study (*p* < 0.05, Figure 5A). For the functions of fungi, coffee litter mulching significantly reduced the abundance of mycorrhizal by 10.40% (*p* < 0.05, Figure 5B). Coffee pericarp mulching significantly increased the abundance of mycorrhizal by 13.42% but significantly reduced the abundance of pathogens by 11.88% in this study (*p* < 0.05, Figure 5B).

### 3.5 Correlation between soil properties and microbial diversity under coffee pericarp and litter mulching

**Figure 6:**
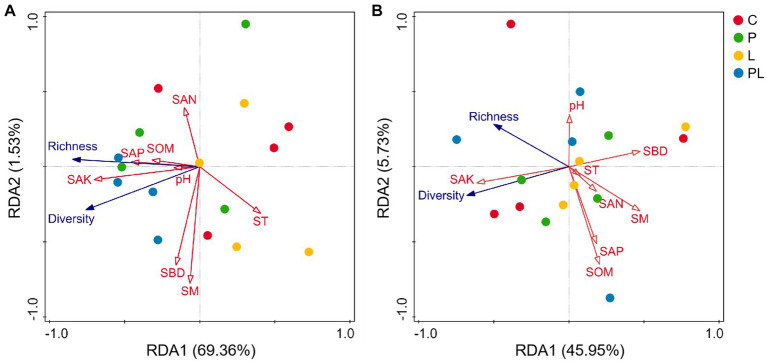
Ordination plots of the results from the redundancy analysis (RDA) to identify the relationships among the soil properties (red arrows) and bacterial **(A)** and fungal **(B)** taxa (blue arrows) at the phylum level. See Figure 1 for treatment abbreviations.

Canonical correlation analysis (RDA) was used to analyze the relationship between soil physicochemical properties and soil microbial (bacterial and fungal) diversity. The results showed that SAK (*F* = 7.30, *p* = 0.02) was the significant soil index in affecting the dominant bacteria. All soil properties variables explained 70.89% of the variation of bacterial richness and diversity among bacterial samples. The first two ranking axes of RDA explained 69.36 and 1.53% of the total variance, respectively (Table 3; Figure 6A). The fungal community was not sensitive to the changes in soil physicochemical indicators, although all soil variables together explained 51.68% of the variation of fungal richness and diversity among fungal samples (Table 3; Figure 6B).

**Table 3:** Ordination plots of the results from the redundancy analysis (RDA) to identify the relationships among the soil properties and microbial (bacterial and fungal) richness and diversity.

Sequences	Bacterial			Fungi		
	Explains	*F*-value	*p*-value	Explains	*F*-value	*P*-value
SAK	34.30	7.30	0.02	13.60	2.20	0.16
SAP	7.20	1.60	0.22	2.40	0.30	0.69
SOM	3.50	0.80	0.39	3.20	0.50	0.50
SAN	5.80	1.30	0.27	2.80	0.40	0.60
pH	8.80	2.20	0.16	3.60	0.50	0.54
SM	9.80	2.90	0.12	3.20	0.50	0.60
ST	1.30	0.40	0.60	3.30	0.40	0.59
SBD	0.30	0.01	0.84	9.50	1.60	0.20

### 3.6 Correlation between soil properties and microbial structure under coffee pericarp and litter mulching

**Figure 7:**
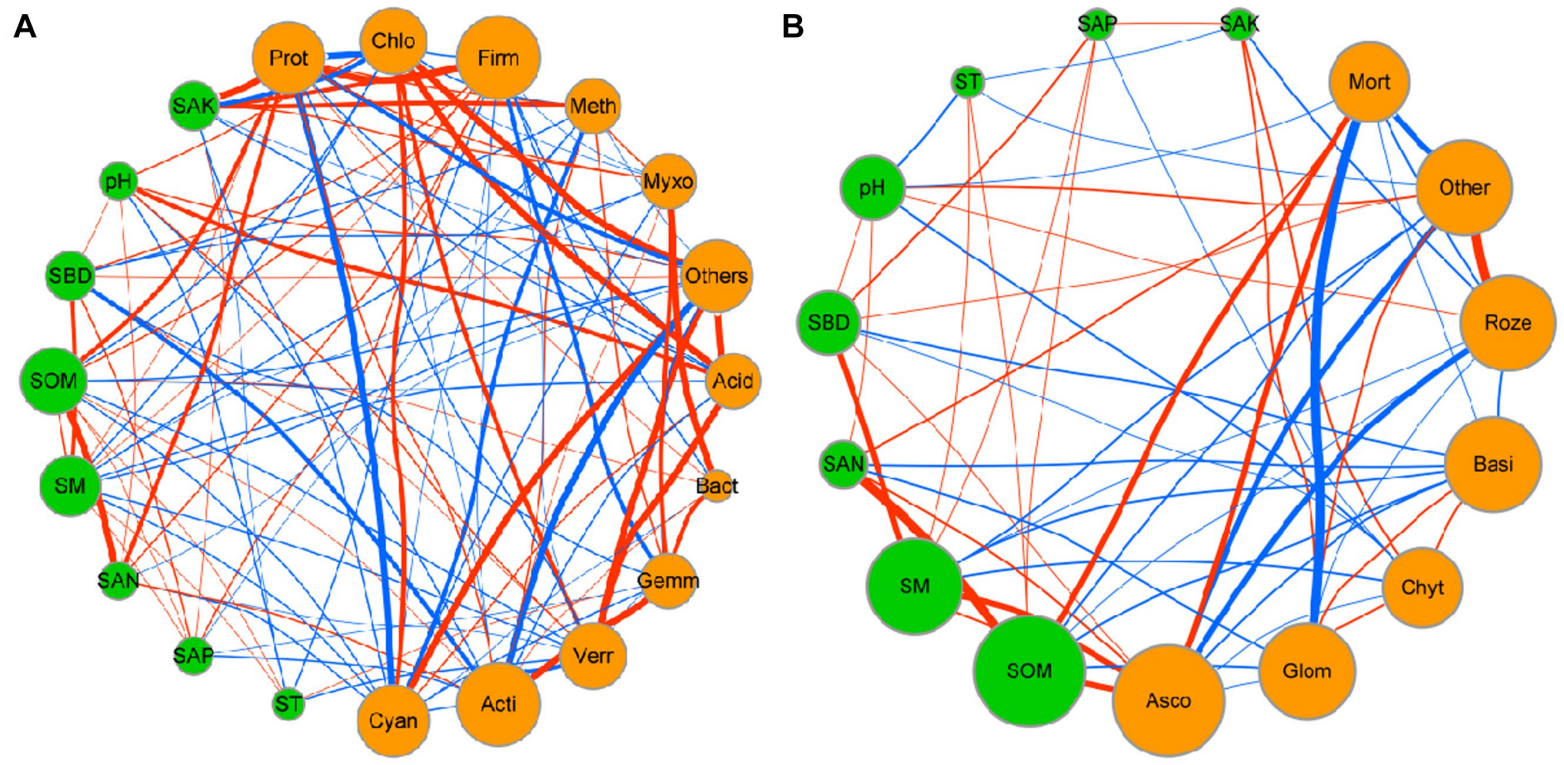
Network interaction diagram of soil properties and bacterial **(A)** and fungal **(B)** taxa at the phylum level. Red lines indicate a positive correlation, while blue lines indicate a negative correlation. The thickness of the line represents the correlation size. The size of the points represents the magnitude of relative types and contents of volatile components.

Network analysis was used to determine the co-occurrence patterns of soil properties and microbial composition based on strong and significant correlations (Figure 7). Overall, different mulch patterns showed a remarkable effect on association networks of soil properties and bacteria at the phylum level. APL (average path length) represents the overall connectivity among nodes in the network, and a smaller value indicates a tighter overall connection. *avgK* (average degree) being higher signifies more connections between nodes in the network. The clustering coefficient is a measure of the degree of connectivity between neighboring nodes of a particular node, and its value ranges from 0 to 1. A higher value of *avgCC* (average clustering Coefficient) implies a closer connection between nodes in the network. Graph density describes the tightness of connections between nodes in the network and ranges from 0 to 1. A graph density closer to 1 indicates a tighter connection between nodes, while a graph density closer to 0 suggests sparser connections between nodes. The values of APL, *avgK*, *avgCC*, and graph density in these empirical networks were 1.03, 14.63, 0.98, and 0.98, respectively (Table 4). More negative co-occurrence relationships were shown between soil properties and bacteria. More negative co-occurrence relationships are shown from soil properties to bacteria in the network graph (Figure 7A), indicating that the change in soil properties had a significant impact on the composition of the bacterial community. Near neutral co-occurrence relationships were shown between bacteria in the network graph, indicating that there was a balance relationship between the dominant species of bacteria. For fungal communities, the values of APL, *avgK*, *avgCC*, and graph density in these empirical networks were 1.97, 4.15, 0.59, and 0.35, respectively (Table 4). More negative co-occurrence relationships were shown between soil properties and fungi. More negative co-occurrence relationships were shown for soil properties to dominant fungal species in the network graph, indicating that the change of soil properties had a similar trend to bacteria in affecting the composition of the fungal community (Figure 7B). More negative co-occurrence relationships were shown between fungi in the network graph, indicating that competition rather than synergy among dominant fungal species in the current study (Figure 7B). As mentioned above, the partial least squares path model was used to quantify the specific causal relationship from the overall perspective and it indicated that the improvement of soil properties was not conducive to maintaining the dominant position of dominant bacterial and fungal species in the microbial community (i.e., coffee pericarp and litter mulching could promote the reproduction of rare species by improving soil properties).

**Table 4:** Topological properties of co-occurring soil and microbial (bacterial and fungal) components networks obtained in several mulch patterns.

Network metrics	Bacteria	Fungi
Number of nodes	20	15
Number of edges	114	58
Number of positive correlations	45	61
Number of negative correlations	56	74
Percentage of the positive link (P%)	44.55	37.78
P% from soil properties to microbial components	39.13	39.29
P% between microbial components	49.09	35.29
Average connectivity (*avgK*)	14.63	4.15
Average clustering coefficient (*avgCC*)	0.98	0.59
Average path length (APL)	1.03	1.97
Graph density	0.98	0.35

## 4 Discussion

### 4.1 Regulation mechanism of different coffee mulches on the soil microenvironment

The effects of ecological cycling cultivation on the soil microenvironment are quite different, and the difference is mainly closely related to the nature of the mulch in different farmland ecosystems (De Godoy Fernandes et al., 2021). Soil physicochemical properties are also affected by differences in the decomposition characteristics of different mulching materials and their regulation of the soil microenvironment (Pavlu et al., 2021). Coffee pericarp mulching significantly increased the content of SAK and SAN in the soil (Figure 1), mainly due to the high content of nitrogen and potassium in the coffee pericarp in this study (Shemekite et al., 2014) and the leaching and migration of nitrogen and potassium into the soil during coffee pericarp decomposition (Mutshekwa et al., 2020). Previous studies indicated that agricultural wastes with a lower ratio of carbon and nitrogen decomposed faster at the initial stage of decomposition (Martinez-Garcia et al., 2021). Coffee leaf litter with high nitrogen content may have a pulse effect on the improvement of soil nutrient content at the initial stage of mulching, while pericarp with low nitrogen content may maintain sustained nutrient release during long-term decomposition (Wang et al., 2016). The weight loss rate (i.e., decomposition rate) of coffee peel was lower than that of litter in this study (Supplementary Figure S1), confirming the previous research results. Therefore, in the late stages of coffee litter and pericarp decomposition, the pericarp cover still tended to increase the soil organic matter content, but the coffee litter had almost no effect on the soil nutrient content. Interestingly, soil temperature tends to decrease under the pericarp mulch treatment (Figure 1), possibly due to the pores formed after the pericarp stacked, which weakens the warming effect of radiation on the soil surface by changing the surface airflow dynamics (Gholamhoseini et al., 2019), while the reduced soil temperature may have a negative feedback effect on the pericarp decomposition, further slowing the rate of pericarp decomposition (Sariyildiz, 2008).

### 4.2 Regulation mechanisms of soil microbiome diversity by different coffee mulch

The soil microbiome is an important component of the farmland ecosystem (Jurburg et al., 2020) and is mainly involved in ecological processes such as the decomposition of organic matter, humus formation, and nutrient cycling in soil (Yadav et al., 2021). Previous studies indicated that agricultural waste mulching regulates the soil microbiome mainly via its own nutrient release and influence on the surface soil microenvironment (Barel et al., 2018). On the one hand, soil nutrient content is an important factor in regulated microbial composition and diversity (Kang et al., 2021). Straw mulch can provide carbon sources and nutrients to the soil microbiome, which has feedback effects on the composition and diversity of the soil microbiome (Bu et al., 2020; Li et al., 2021). Previous studies have shown that the increase of soil nitrogen and phosphorus content can increase the ratio of phospholipid fatty acids between bacteria and fungi (Leff et al., 2015; Ling et al., 2017), and mulch treatment can promote the abundance of some functional microbial genes and increase bacterial community diversity by improving soil nutrient content (Muhammad et al., 2021). The soil potassium content rather than nitrogen was the main factor affecting soil bacterial richness and diversity (Zheng et al., 2022), which may be related to the specific demand of coffee for potassium. Potassium is important for the recruitment and proliferation of soil bacteria (Dong et al., 2019), and pericarp mulching had a positive effect on soil microbial richness and diversity by increasing soil potassium content in the present study (Figure 6). On the other hand, soil temperature, moisture, and soil bulk density are also the main factors affecting soil bacterial diversity (Rahman et al., 2021). However, bacterial diversity did not respond to changes in soil moisture as well as pH in this study (Table 3; Figure 6A), indicating that the above soil physicochemical and microenvironment were not the main factors driving the bacterial community under different coffee mulches in the test site.

### 4.3 Regulation mechanism of soil microbiome structure by different coffee mulch

Soil microbial composition and structure under different agricultural waste mulch are synergistically regulated by allelochemicals, soil properties, and the microenvironment (Castellano-Hinojosa and Strauss, 2020). For bacteria, on the one hand, different waste qualities and their decomposition characteristics are the important influencing factors (Yan et al., 2018; Zhai et al., 2019) Previous studies have shown that *Proteobacteria* is the dominant bacterial phyla in soil (Kim et al., 2021), which has a wide range of physiological and metabolic functions and the ability to adapt to a variety of complex environments. Coffee pericarp cover provides sufficient metabolic substrate to promote the growth and reproduction of *Proteobacteria* as they can multiply rapidly in a carbon-rich environment (Lauber et al., 2008). *Firmicutes* can promote the decomposition of soil organic matter, and their abundance was positively correlated with soil organic matter available potassium and nitrogen content in a previous study (Castellano-Hinojosa and Strauss, 2020). Coffee pericarp mulching cultivation increased the abundance of pachyderma by increasing the content of SAK and SAN content in this study. *Cyanobacteria* are a kind of bacteria that can obtain energy through photosynthesis, and a small proportion of them are also heterotrophic bacteria (Soo et al., 2019). Coffee mulch increased soil heterotrophic metabolic substrates, significantly enhancing the competitive advantage of heterotrophic microorganisms, and causing a significant decrease in the relative proportion of *Cyanobacteria*. On the other hand, the difference in soil properties and hydrothermal conditions are the important factors affecting bacterial composition and structure. There was a negative correlation between soil properties and the dominant bacterial phyla, indicating that the increase in soil nutrients under coffee pericarp mulching is beneficial to the recruitment and proliferation of rare bacteria rather than the dominant bacterial phyla, which has a positive effect on improving the assembly of resistant structures in soil bacterial communities. Moreover, the tendency toward neutral correlations between dominant bacterial phyla suggests that coffee pericarp mulching is advantageous to maintain the relative stability of the dominant bacterial microbiota structure.

For fungi, soil fungal communities in this study were not sensitive to the mulching of coffee waste, except *Ascomycota* and *Basidiomycota*. *Ascomycota* is one of the main fungi that decompose cellulose and lignin (Heeger et al., 2021). The high content of cellulose in the residue of petioles and dead branches in the late stages of coffee litter may have caused an increase in the relative proportion of *Ascomycota* (Supplementary Figure S1). The dominant fungal phyla may be less sensitive to changes in soil properties than bacteria, although there were also many negative correlations between the soil properties and the dominant fungal phyla. Most notably, more negative correlations between fungal dominant phyla under coffee mulch indicated that the competitive relationships among fungal dominant phyla outweighed the synergistic effects (Figure 7B). Coffee pericarp mulching might adjust fungal community structural assembly and succession direction by intensifying fungal competition for soil niches.

### 4.4 Effects of different coffee mulch on soil microbiome functions

It is generally believed that microbial community composition is closely related to microbial function (Jiang and Zeng, 2022). For example, microbial composition significantly regulates substrate use efficiency (i.e., main indicators of microbial decomposition function), especially in tropical soil (Bonner et al., 2018). Both coffee pericarp and litter mulching significantly promoted the abundance of heterotrophic bacteria and bacteria involved in the nitrogen cycling and decreased the abundance of autotrophic bacteria in this study (Figure 5A), which may be caused by exogenous nutrient input (Yang et al., 2022). Interestingly, the decreased abundance of bacteria involved in organic matter degradation under coffee litter mulching may be related to the decomposition characteristics of coffee leaves as the organic matter that coffee litter releases during the later stage of decomposition was not conducive to the proliferation of organic matter degrading bacteria in the current study. The increase of mycorrhizal fungi and decrease in pathogenic fungi under coffee pericarp mulch treatment indicated that coffee pericarp enhanced the antagonistic effect of coffee plantation soil against harmful microorganisms by enhancing nutrient supply, and had a positive effect on the healthy growth of coffee plants. The decrease of mycorrhizal fungi under coffee litter mulch treatment may have a slightly negative effect on nutrient uptake and stress resistance in coffee plants.

## 5 Conclusion

Coffee litter mulching has little impact on bacterial community abundance and diversity, whereas coffee pericarp mulching promotes the richness and diversity of soil bacterial community by increasing the content of available potassium and nitrogen content, increasing the relative abundance of *Proteobacteria* and *Firmicutes*, and reducing the relative abundance of *Cyanobacteria*. Coffee pericarp mulching rather than litter mulching promotes the function of anoxygenic photoautotrophy, nitrogen respiration, nitrate respiration, nitrite respiration, and denitrification of bacteria, while soil fungal communities are not sensitive to the response of different coffee mulch treatments. Coffee pericarp increases soil microbiome diversity and maintains the stability of structure and function via releasing and transporting nutrients to the soil. The use of an ecological recycling pattern for coffee cultivation is conducive to conserving soil health and promoting sustainable development of related industries in coffee plantations.

## Data availability statement

The datasets presented in this study can be found in online repositories. The names of the repository/repositories and accession number(s) can be found below: NCBI – http://data.mendeley.com/datasets/kzdg45x84s/1.

## Ethics statement

The field research occurred on the plantations in Hainan Province with approval from the Garden Supervisor or Planters.

## Author contributions

SZ: Conceptualization, Data curation, Visualization, Writing – original draft, Writing – review & editing. AZ: Funding acquisition, Resources, Writing – review & editing. QZ: Conceptualization, Writing – review & editing. YZ: Investigation, Writing – review & editing. DW: Writing – review & editing. LS: Writing – review & editing. XL: Writing – review & editing. YS: Writing – review & editing. LY: Writing – review & editing. XW: Writing – review & editing. NA: Writing – review & editing. YD: Writing – review & editing. JT: Writing – review & editing. YL: Writing – review & editing. ZL: Writing – review & editing. LL: Writing - review & editing.
