# Supplementary material for: Effects of coffee pericarp and litter mulsching on soil microbiomes diversity and functions in a tropical coffee plantation, South China
Source: Front Microbiol. 2024 Jan 8;14:1323902. doi: 10.3389/fmicb.2023.1323902 (PMC10800520; doi:10.3389/fmicb.2023.1323902)
Supplement: Supplementary file 1 [file Data_Sheet_1.docx]

**Figure S1** Effects of coffee pericarp and litter mulch patterns on coffee waste decomposition. P represents coffee pericarp, L represents coffee litter, and PL represents coffee pericarp mixed with litter. Different letters represent significant differences between treatments (*P* < 0.05).

**
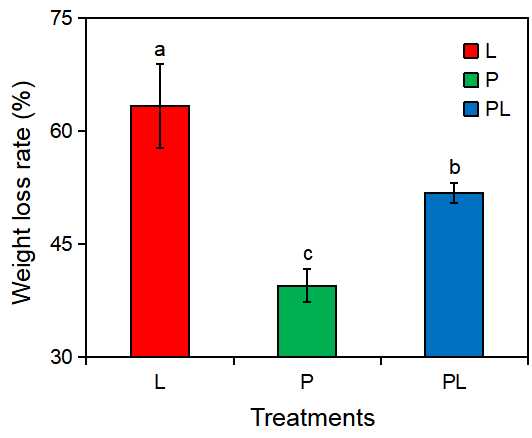
**
